# In this month’s Bulletin

**DOI:** 10.2471/BLT.26.000626

**Published:** 2026-06-01

In the editorial section, Joseph Millum et al. (370) argue for the need to embed ethics in health research priority-setting. Zhuo Li et al. (371) present a case for using spatial evidence in waterborne disease control.

## Burkina Faso, Ethiopia, Kenya and Malawi

### Implementation of the global action plan for wasting

Adriana Newman et al. (417–426) list geographic priorities.

## Gaza Strip 

### Maternal and neonatal outcomes 

Zeina Jamaluddine et al. (386–395) follow a cohort of pregnant women during conflict.

## Guatemala and India

### Use of the International Guide for Monitoring Child Development

Scott Tschida et al. (396–406) estimate costs of adaptation.

## WHO European Region

### Governance of artificial intelligence in the health system

Keyrellous Adib et al. (374–385) analyse national provisions.

## Global

### Interventions to reduce antimicrobial use in livestock

Fiona Emdin et al. (407–416) review and map the evidence.

### Cross-border telehealth services

Caroline Perrin Franck et al. (427–434) describe a policy analysis and expert consultation.

### Next steps for Chagas disease control

Debbie Vermeij et al. (435–437) detail elimination requirements.

### Tuberculosis prevention and care 

Kerri Viney et al. (438–440) seek equitable provision for vulnerable populations.

